# The impact of community-based health insurance on health service utilization and financial risk protection in Ethiopia

**DOI:** 10.1186/s12913-022-09019-6

**Published:** 2023-01-23

**Authors:** Yibeltal Kiflie Alemayehu, Ermias Dessie, Girmay Medhin, Negalign Birhanu, David R. Hotchkiss, Alula M. Teklu, Mizan Kiros

**Affiliations:** 1 MERQ Consultancy PLC, Addis Ababa, Ethiopia; 2 World Health Organization – Ethiopia, Addis Ababa, Ethiopia; 3 Department of Health Policy and Management, Institute of Health, Jimma University, Jimma, Ethiopia; 4 School of Public Health and Tropical Medicine, Tulane University, New Orleans, USA; 5 Ministry of Health, Addis Ababa, Ethiopia

**Keywords:** Community-based health insurance, Health care utilization, Health financing, Financial risk protection, Ethiopia

## Abstract

**Background:**

Evidence on the effectiveness of community-based health insurance (CBHI) in low-income countries is inconclusive. This study assessed the impact of CBHI on health service utilization and financial risk protection in Ethiopia.

**Methods:**

We conducted a comparative cross-sectional study nested within a larger national household survey in 2020. Data was collected from three groups of households—CBHI member households (*n* = 1586), non-member households from CBHI implementing woredas (*n* = 1863), and non-member households from non-CBHI implementing woredas (*n* = 789). Indicators of health service utilization, out-of-pocket health spending, catastrophic health expenditure, and impoverishment due to health spending among CBHI members were compared with non-members from CBHI implementing woredas and households from non-CBHI implementing woredas. Propensity score matching (PSM) was used to account for possible selection bias.

**Results:**

The annual number of OPD visits per capita among CBHI member households was 2.09, compared to 1.53 among non-member households from CBHI woredas and 1.75 among households from non-CBHI woredas. PSM estimates indicated that CBHI members had 0.36 (95% CI: 0.25, 0.44) and 0.17 (95% CI: -0.04, 0.19) more outpatient department (OPD) visits per capita per year than their matched non-member households from CBHI-implementing and non-CBHI implementing woredas, respectively. CBHI membership resulted in a 28–43% reduction in annual OOP payments as compared to non-member households. CBHI member households were significantly less likely to incur catastrophic health expenditures (measured as annual OOP payments of more than 10% of the household’s total expenditure) compared to non-members (*p* < 0.01).

**Conclusion:**

CBHI membership increases health service utilization and financial protection. CBHI proves to be an important strategy for promoting universal health coverage. Implementing CBHI in all woredas and increasing membership among households in woredas that are already implementing CBHI will further expand its benefits.

**Supplementary Information:**

The online version contains supplementary material available at 10.1186/s12913-022-09019-6.

## Background

The pledge of nations around the world to ‘leave no one behind’ as a cross-cutting principle of the Sustainable Development Goals, reaffirms global commitment to Universal Health Coverage (UHC) [1, 2]. UHC, an essential yet challenging target, involves ensuring that all people receive quality health services without being exposed to financial hardship in paying for the services. This ambition presents a challenge to developing countries where out-of-pocket health expenditure represent a key source of revenue for health systems [3, 4]. In these settings, promoting UHC requires mobilizing adequate financial resources to expand access to essential health services on the one hand and protecting health service users from financial hardship by minimizing out-of-pocket (OOP) payments on the other hand [5].

Ethiopia recorded substantial improvements in health outcomes over the past two decades [6, 7]. Improvements in access to and utilization of health services have been among the major contributors to these achievements [8–14].

During this period, expansion of health services has been accompanied by substantial increases in health expenditure. Between 1996 and 2017, total health expenditure (THE) increased from US$230.1 million to US$3.1 billion; and annual per capita spending on health care increased from US$4.1 to US$33.2 [15]. However, average per capita health spending in Ethiopia is still far below the World Health Organization’s recommended US$86 per person needed to secure essential health care services in low-income countries [16]. OOP payments also constitute an unacceptably high proportion of THE (31% in 2017) as opposed to the recommended rate of 15–20% to ensure protection of households from catastrophic and impoverishing health expenditures [5, 15]. In addition to financial risks, the cost of medical care has also been identified as an important barrier to health service utilization in Ethiopia [6]. To address these challenges, the Government of Ethiopia has been implementing a series of healthcare financing reforms over the past two decades.

Community-based health insurance (CBHI) was piloted between 2011 and 2014 as one of the healthcare financing initiatives of Ethiopia. The objectives of CBHI include removing financial barriers, reducing catastrophic out-of-pocket payments, increasing health services utilization, improving quality of care, enhancing health equity, and enhancing sustainable health financing through mobilization of domestic resources [17]. The initiative was piloted in 2011 and scaled up in 2015 [18] reaching more than 80% of woredas (equivalent to districts) in 2020. In CBHI implementing woredas, 49% of eligible households have subscribed; of the total members, 78.8% were paying members while the remaining 21.2% were indigents [19–21].

Investigation and documentation of the impact of CBHI on health service utilization and financial risk protection through rigorous study would facilitate informed decisions on healthcare financing mechanisms and advocate for CBHI program expansion. In Ethiopia, evidence from previous studies in general remained inconclusive because of methodological limitations, small geographic coverage, and focus on pilot implementations that limited the generalizability of findings [22–26]. The objective of this study was therefore to assess the impact of CBHI membership on health service utilization and financial risk protection among CBHI members in Ethiopia. We compared health service utilization, the incidences of catastrophic health expenditure and impoverishment due to out-of-pocket payments among CBHI member households with those of non-members.

## Methods

### Context

Ethiopia is the second most populous nation and the fastest growing economy in Africa, with an estimated population of 114.9 million in 2020 and an average economic growth rate of 9.8% between 2009 and 2019. Per capita income of Ethiopians is US$855 making the country one of the world’s low-income countries [27–29]. More than three fourth of the population (78.3%) lives in rural areas with scattered population settlement patterns where subsistence farming and animal husbandry are the main sources of livelihood [30]. The country is administratively divided into 10 regional states and two city administrations. Regions and cities are divided into woredas (equivalent to districts) which are further divided into Kebeles, which represent the lowest administrative structure serving an average of 5,000 population.

Health service delivery in Ethiopia is predominantly public. The public sector is organized in a three-tier healthcare delivery model that includes primary level care provided by primary healthcare units (composed of health centers and health posts) and primary hospitals, secondary level care provided by general hospitals, and tertiary level care provided by specialized hospitals [31]. Healthcare is financed predominantly by external funding (35%), government expenditure (32%), and households’ out-of-pocket payments (31%). The role of health insurance has been insignificant until recently [15].

### Study design

A comparative cross-sectional study design was used to assess the impact of CBHI on health services utilization and financial risk protection among CBHI member households in the regions of Tigray, Amhara, Oromia, and Southern Nations Nationalities and Peoples (SNNP), where CBHI has been implemented for the longest period in the country. The study was nested within a national study that was designed to assess willingness and ability of households to pay for CBHI, at the national level. The main study collected data from all regions of the country, irrespective of CBHI implementation status. For the current analysis (analysis of the impact of CBHI), we used data collected from regions where the CBHI has been implemented. Health service utilization and the incidences of catastrophic and impoverishing health expenditures were measured and compared between CBHI member households and non-members from two categories of districts in the regions where CBHI has been implemented: CBHI implementing and non-CBHI implementing districts.

### Study participants

Three groups of households were established for the household survey based on the implementation of CBHI at the district level and membership status at the household level. Our primary sampling unit was the enumeration area (EA), a geographically defined cluster of about 100–150 households in rural areas and 150–200 households in urban areas, developed and maintained by the Central Statistical Agency of Ethiopia for census and survey purposes. The sampling unit for second stage sampling was the household. Heads of households and their spouses were eligible respondents for the survey.

### Sample size estimation

The sample size for the national survey on willingness and ability to pay was estimated assuming that 50% of the households would be willing to pay the financial contribution for CBHI with 95% confidence and 2.5% margin of error. This yielded an initial sample size of 1,537 households. The plan to use a multistage sampling strategy required adjusting the sample size for possible clustering at different levels. The design effect is a function of the number of clusters, the number of households to be selected from each cluster, and intraclass correlation. Intraclass correlation was calculated for the values of the wealth index using the data collected during the National Assessment of the Health Extension Program in Ethiopia. The resulting estimate of the intraclass correlation was 0.12746. After comparing the travel costs of adding more clusters with the costs of adding more households within a cluster, including the impact of increasing the design effect on the total sample size, the optimum combination of the number of clusters or enumeration areas (EAs) and the number of households within a cluster was found to be 166 and 34, respectively, resulting in a design effect of 5.2. Increasing the number of households by two in every EA to take account of non-response, our final sample size was 5976 households from 166 EAs. From these, 118 EAs were allocated to regions implementing CBHI. Our analysis of the impact of CBHI was based on this sub-set of the national survey data. Before the conduct of the study, we estimated power to estimate the impact of CBHI based on this subset of data for two scenarios for selection of comparison households: (a) non-CBHI members are recruited from CBHI woredas, (b) non-CBHI members are recruited from non-CBHI woredas. Under scenario (a) the study had a power of 89–100% to detect mean difference of 0.67 in OPD visit per capita per year, or 3.0% in treatment seeking for a recent illness, or ETB 234 in OOP health expenditure per capita, or 3.0% in the incidence of catastrophic expenditure. Similarly, under scenario (b) the study had a power of 76–100% to detect mean differences of 0.47 in OPD visit per capita per year, or 12.0% in treatment seeking for a recent illness, or ETB 362 in OOP health expenditure per capita, or 5.0% in incidence of catastrophic expenditure.

### Sampling

Study households were identified through a two-stage stratified sampling method. Enumeration areas in the four study regions were first stratified into CBHI implementing and non-CBHI implementing categories based on administrative records of the authority responsible for overseeing insurance schemes. These two categories supplemented with other regions not yet implanting CBHI were further stratified by the major livelihood of the woreda (urban, rural and pastoralist). This resulted in a total of 27 strata that have at least one EA. The total of 166 EAs were allocated to these 27 strata proportional to the population size of each stratum. Within the four CBHI implementing regions in which the impact study was conducted, the share of EAs were 96 EAs (81 rural and 15 urban) from the CBHI implementing stratum and 22 (11 rural and 11 urban) from the non-CBHI implementing stratum. For each selected EA, a complete list of households was constructed to generate a sampling frame of CBHI member and non-CBHI member households. Then, 36 households were randomly selected from each EA using computer-assisted random sampling technique. In CBHI implementing EAs, the sampling was stratified to include 18 member and 18 non-member households. In EAs where the total numbers of members or non-members was less than 18, all available households in that category were taken and the remaining portion of the sample size would be added to the other category so that 36 households are included in total. This resulted in 1586 CBHI members, 1863 non-CBHI members from CBHI-implementing districts, and 789 non-CBHI members from non-CBHI-implementing districts.

### Measurement

Health service utilization was measured in the forms of probability of modern healthcare seeking for the most recent episode of illness during the one-month period preceding the survey and per capita health facility visits (for curative as well as health promotion and disease prevention services such as antenatal care and immunization) during the last one month. Financial risk protection was measured using out-of-pocket health expenditure, the incidences of catastrophic health expenditure and impoverishment due to out-of-pocket health expenditure.

#### Probability of modern healthcare seeking at time of illness

The proportion of household members with at least one episode of illness during the one-month period preceding the survey who sought health service from a modern healthcare provider (public or private) for their most recent illness.

#### Per capita health facility visits

The average number of health facility visits that a household member had during the one-month period preceding the survey (visits for any type of health services, including curative, follow up, and health promotion services).

#### Out-of-pocket health expenditure

Payments made by a household at the point they receive health services.

#### Catastrophic health expenditure

A household is classified as having catastrophic health expenditure if the total OOP health payment equals or exceeds 10% of the total household expenditure. Our conclusions and discussions are based on this threshold, but we also included other thresholds such as 25% of the total expenditure and 40% of non-food expenditure for future reference. We chose the “10% of total households expenditure threshold” since it is the nationally used threshold for catastrophic health expenditure. For example, MoH-Ethiopia and Ethiopia Health Insurance Service puts their five years (2021–2025) financial risk protection targets based on the 10% cut-off point. In addition, the sustainable Development Goals UHC target used the same threshold. Our analysis was based on the detail computation technique discussed by Wagstaff, eat al. [32].

#### Impoverishment due to out-of-pocket health spending

A non-poor household is impoverished by health payments when it becomes poor after paying for health services. Impoverishment due to health payments was measured in terms of absolute increases in poverty headcount, poverty gap, and normalized poverty gap after OOP health payments. Wagstaff et al. described the detail methods on how to compute poverty head count, poverty gap and normalized poverty gap [32]. The 2015/16 national poverty line (7 184 Ethiopian Birr per capita) [33] was adjusted for general food and non-food inflation based on consumer price indices reported by the Central Statistical Agency for the years 2015/16 to 2019/20. The resulting adjusted poverty line for 2019/20 was 10 053 birr per capita. This poverty line was used to calculate the poverty headcount and the poverty gap and determine the differences in poverty indices before and after health payments.

### Data collection tools and data collectors

Data were collected by experienced and trained data collectors using standard questions adapted from related studies in the fields of health service utilization and household consumption surveys. All data collection tools were translated from English to the major local languages spoken in the study regions. Translated versions were back-translated into English for quality assurance purposes. All the tools were pre-tested in similar settings prior to data collection. In areas where languages other than the major local languages are spoken, mainly in SNNP region, translators assisted data collectors.

### Data collection

Data were collected through household and market surveys. Household data was collected through face-to-face interviews with heads of households and their spouses and measurement of food items using weight scales. Data on household’s general characteristics including family size and composition, health service utilization patterns, and household food and non-food consumption and expenditure, including OOP health payments were collected through two rounds of household visits. Food consumption data was collected for a recall period of three days in the first round and four days in the second round.

Consumption of own products was valuated using data collected on local market price through local market surveys. Price data from the market survey was used in the consumption aggregate to determine the level of prices for various items in local markets in the study area and allowing for estimation of monetary values for items produced and consumed at home. Open markets, kiosks, groceries, butcheries, pharmacies, supermarkets, and other service establishments where households in the EAs purchase most of their goods and services for household consumption and other purposes were used as sources of data.

Household and market survey data were collected using the electronic Census and Survey Processing System (CSPro) with pre-designed data quality assurance features loaded on Android devices. The program included features such as assignment of data collection tools to data collection sites, listing of households in study EAs, random sampling of households within EAs, collection of household and market data, synchronization of data with supervisors, and online submission of data to a central server. The system was prepared with appropriate sequencing of questions and data validation rules to ensure the collection of valid and consistent data.

### Data management and analysis

Data collected using CSPro were synchronized to supervisors’ accounts on a daily basis. Supervisors then uploaded quality-checked records to a central server. A central data quality assurance team checked the quality of submitted data on a daily basis. Upon completion of data collection fieldwork, data submitted to the central server was compared with data on individual tablet computers to ensure complete synchronization. The final dataset was cleaned to remove duplicate records and exported to Stata version 16.0 for Windows for analysis. Consumption data collected from households was combined with price data collected through market surveys to create consumption aggregates. Individual-level data collected about members of households were aggregated to household-level variables.

The distribution of outcome variables (health service utilization and health expenditure), exposure variables (CBHI implementation status of woredas and membership of households), and covariates (socio-demographic and other characteristics of households) were summarized using descriptive statistics. The effect of CBHI was estimated by comparing outcomes between CBHI member households and the two categories of comparison groups: 1) non-member households from CBHI-implementing districts and 2) households from non-CBHI-implementing districts. Sampling weights, calculated as the inverse probability of the selection of sampling units, were used while analyzing household data.

We used propensity score matching to estimate the effect of CBHI by accounting for possibilities of bias arising from voluntary enrolment in CBHI. Propensity scores were estimated using the logit model regressing enrollment in CBHI on household level covariates, including household head’s characteristics such as age, sex, education, marital status, literacy, religion, and occupation; socioeconomic status measured as the aggregate household annual expenditure; chronic illness among members (a disease that lasts more than 3 months) calculated as the number of members having at least one chronic disease in a household divided by household size; and self-reported health status of household members on a five-point scale (1 = very good to 5 = very bad). Model outputs are presented in Table 3 and Table 6 of the linked supplementary file. We used the nearest neighbor matching method, which matched each CBHI member to a comparison household with the closest propensity score. We ran two separate models to estimate the impact of CBHI membership. In model 1, we used non-CBHI members from CBHI-implementing woredas as a comparison group, and in model 2, we used households from non-CBHI implementing woredas as a comparison group. There was good level of matching in both models; the proportion of observations on support was 92.9% among the non-treated and 97.2% among the treated, in model 1; and it was 82.1% among the non-treated and 80.7% among the treated, in model 2. Detailed common support statistics are presented in Table 1 and Table 4 of the linked supplementary file. The average treatment effect on the treated (ATT), the effect of CBHI membership among CBHI members, was calculated as the average difference between matched pairs of households. The distribution of the propensity scores matched satisfactorily between CBHI members and non-members in both models (See supplementary file for quality of matching).

### Ethics approval and consent to participate

The study was conducted in accordance with applicable ethical standards. The study protocol was reviewed and approved by the Institutional Review Board of the Ethiopian Public Health Institute (Ref No: EPHI 613/624 dated 18 February 2020). Official permissions were obtained from relevant authorities at different levels. Verbal informed consent was obtained from all participants after they were provided adequate information about the study.

## Results

### Socio-demographic characteristics of study households

Data was collected from 4 238 households; these include 1 586 (37.4%) CBHI members, 1 863 (44.0%) non-CBHI members from the CBHI-implementing woredas, and 789 (18.6%) households from non-CBHI-implementing woredas. The majority of study households were from rural areas (69%) and male-headed (77%). A higher proportion of household heads among CBHI members had no formal education, and were unmarried compared to non-members. Similarly, CBHI member households were poorer, had larger family sizes, and had a larger share of elders (Table 1).

**Table 1:** Socio-demographic characteristics of study households by CBHI membership status

Variables	Categories	CBHI members (%)	Non-members from CBHI woredas (%)	Non-members from non-CBHI woredas (%)	Total (%)
***Unweighted number of HHs***		***1,586***	***1,863***	***789***	***4,238***
Sex of head of household	Female	20	23	28	23
	Male	80	77	72	77
Age (in years) of head of household	< 35	26	46	42	38
	36–45	28	23	23	25
	46–64	32	21	24	26
	≥ 65	14	10	11	12
Formal education of head of household	No formal education	55	47	42	49
	Primary education	24	22	18	22
	Secondary and above	21	31	41	29
Occupation of head of household	Unemployed	14	11	16	13
	Self-employed	79	71	60	72
	Employed (private and gov)	4	14	19	11
	Other occupation	3	4	5	4
Marital status of head of household	Married	79	74	69	75
	Not married	21	26	31	25
Mean household size		5.1	4.30	4.24	4.59
Per capita expenditure quintiles	1st quintile (poorest)	21	16	17	18
	2nd quintile	21	18	17	19
	3rd quintile	24	20	14	20
	4th quintile	19	22	21	21
	5th quintile (richest)	15	24	31	22
Composition of household members	Share of children < 5	11	15	11	13
	Share of elders ≥ 65 years old	7	5	5	5
	Proportion of households with at least one member with a chronic illness	11	8	9	9
Type of place of residence	Rural	77	72	46	69
	Urban	23	28	54	31
Region	Tigray	7	6	13	8
	Amhara	38	29	5	28
	Oromiya	41	42	32	40
	SNNP	14	22	50	25

### Health service utilization

The incidence of self-reported illness to at least one member of the household was 34% for the one-month preceding the study. Self-reported illness was higher among CBHI member households (43%) compared to non-members from CBHI woredas (27%) and households from non-CBHI woredas (37%). The probability of treatment seeking at times of illness was 67%. Treatment seeking during illness was slightly higher among CBHI member households (70%) compared to non-members from CBHI woredas (67%) and households from non-CBHI woredas (58%) (Table 2).

**Table 2:** Incidence of self-reported illness, probability of treatment seeking, and outpatient health facility by CBHI implementation and membership status

	CBHI members	Non-members from CBHI woredas	Non-members from non-CBHI woredas	Total
***Unweighted number of households***	***1,586***	***1,863***	***789***	***4,238***
Proportion of individuals with an illness during the last one month	13	8	12	11
Proportion of households with at least one sick member in the last one month	43	27	37	34
Probability of treatment seeking during illness among households with at least one sick member	70	67	58	67
Proportion of households with at least one OPD visit in the last month	77	72	64	73
Per capita OPD visits per year^a^	2.09	1.53	1.75	1.77
Source of OPD service				
Health center/Health post	58	42	39	48
Government hospital	22	21	22	21
Private health facilities	20	37	39	31
Proportion of households with at least one inpatient admission in the last one year	6.1	8.0	5.7	

Per capita OPD visit per year = per capita OPD visits per month ^a^ 12

Overall, health service utilization, as measured by number of outpatient department (OPD) visits, was 1.77 visits per person per year. CBHI member households had higher annual OPD visits compared to non-member households from both CBHI-implementing and non-CBHI implementing woredas. The annual number of per capita OPD visits among CBHI member households was 2.09 compared to 1.53 and 1.75 among non-member households from CBHI woredas and non-CBHI woredas, respectively. Public health facilities were the predominant sources of OPD visits. Health centers and health posts (primary healthcare units) were more frequently used as a source of outpatient care by CBHI members compared to non-members (Table 2).

**Fig. 1:**
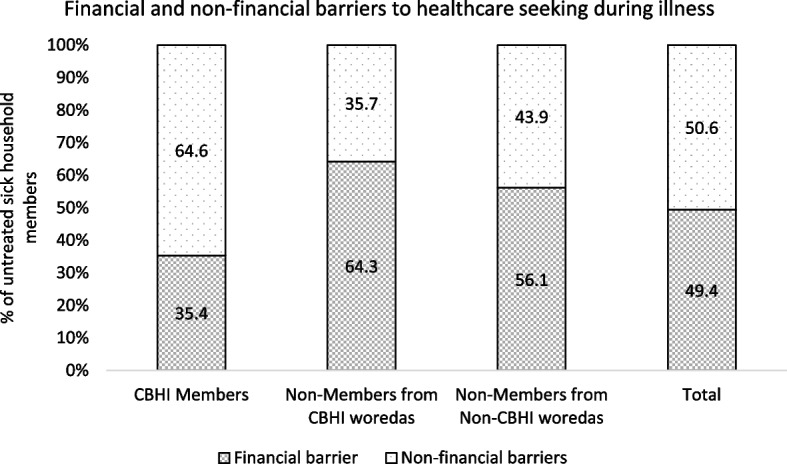
Financial and non-financial reasons for not seeking care during illness

Respondents who reported a sick family member who failed to seek modern healthcare were asked their reasons for not seeking healthcare. Financial barriers, described as either high cost of services or lack of money, was reported as a major reason for not seeking care during illness by half of the respondents. The relative share of financial barrier as a reason for not seeking care was lower among sick members from CBHI member households compared to those from non-CBHI members (Fig. 1).

### Out-of-pocket health expenditure and incidence of catastrophic and impoverishing health expenditures

On average, households spent ETB 451 per person per year for healthcare. A higher share (76.7%) of OOP health payments were related to outpatient services. From the total OOP health payments, 405 (89.8%) was for direct medical expenses. Direct medical expenses were lower among CBHI member households compared to non-member households both for outpatient and inpatient services (Table 3).

**Table 3:** Per capita OOP health payments for outpatient and inpatient services by CBHI implementation and membership status

		**CBHI members**	**Non-members from CBHI woredas**	**Non-members from non-CBHI woredas**	**Total**
Per capita OOP health payment in birr (for outpatient services)	Direct medical	190	365	487	319
	Direct non-medical	28	26	23	27
	Total	218	391	510	346
Per capita OOP health payment in birr (for inpatient services)	Direct medical	71	97	89	86
	Direct non-medical	19	21	14	19
	Total	90	118	103	105
Per capita OOP health payment in birr (total for outpatient and inpatient services)	Direct medical	261	462	576	405
	Direct non-medical	47	47	37	46
	Total	308	509	613	451

The incidence of catastrophic health expenditure was 9.5%, 1.2%, and 3.7% when the thresholds of 10% of total expenditure, 25% of total expenditure, and 40% of non-food expenditure, respectively, were used as thresholds for defining CHE. With the 10% of total expenditure as a threshold, catastrophic health expenditure was 7.8% among CBHI member households, 9.8% among non-CBHI member households from CBHI implementing woredas, and 12.6% among households in non-CBHI woredas (Table 4).

**Table 4:** Incidence of catastrophic health expenditure and impoverishment due to OOP health payments

Measures of catastrophic health expenditure and impoverishment due to OOP health spending		CBHI members	Non-members from CBHI woredas	Non-members from non-CBHI woredas	Total
***Catastrophic and impoverishing health expenditures from OOP health expenditure (including direct medical and direct non-medical expenses)***					
Incidence of catastrophic health expenditure by different thresholds	> 10% of total expenditure	7.80	9.80	12.60	9.50
	> 25% of total expenditure	0.50	1.70	1.30	1.20
	≥ 40% of non-food expenditure	2.50	4.50	3.80	3.70
Impoverishment due to health spending	Absolute increase in poverty headcount	1.90	1.20	3.40	1.80
	Absolute increase in poverty gap (ETB)	67.59	64.16	112.01	73.09
	Absolute increase in normalized poverty gap	0.70	0.60	1.10	0.70
***Catastrophic and impoverishing health expenditures from OOP health expenditure only for direct medical expenses***					
Incidence of catastrophic health expenditure by different thresholds	> 10% of total expenditure	6.40	9.20	11.20	8.50
	> 25% of total expenditure	0.40	1.30	1.30	1.00
	≥ 40% of non-food expenditure	1.40	3.60	3.80	2.80
Impoverishment due to health spending	Absolute increase in poverty headcount	2.30	1.20	3.60	2.00
	Absolute increase in poverty gap (ETB)	82.26	70.32	119.79	82.67
	Absolute increase in normalized poverty gap	0.80	0.70	1.20	0.80

In the overall sample, 26.5% of households were below the national poverty line; the poverty gap was ETB 774.6; and the normalized poverty gap was 29.1% before health payments. Out-of-pocket health payments increased these poverty indices by 1.8%, ETB 73.1, and 0.7%, respectively. Impoverishment due to OOP health payments among CBHI member households was lower compared to households from non-CBHI implementing woredas but higher compared to non-CBHI member households from CBHI-implementing woredas (Table 4).

### The impact of CBHI on health service utilization and financial risk protection

CBHI membership is significantly associated with a higher number of per capita OPD visits while inpatient admission during the one-year period preceding the survey, was lower among CBHI members than non-members. Per capita OPD visit per year among CBHI members was 22.6% and 9.1% higher compared to non-CBHI members from CBHI woredas and non-CBHI woredas, respectively. For every 1000 population from CBHI member households, we expect 360 and 170 more OPD visits every year compared to what we expect from the same number of people from non-member households in CBHI and non-CBHI woredas, respectively (Table 5).

**Table 5:** Estimated effect of CBHI membership on outpatient and inpatient care utilization among CBHI members

Variable	Model^a^	Sample	CBHI members	Non-members	Difference		
					Estimate	95% CI	
						LL	UL
Number of OPD visit per capita per year	Model 1	Unmatched	1.93	1.6	0.33		
		ATT	1.95	1.59	0.36	0.25	0.44
	Model 2	Unmatched	1.93	1.81	0.12		
		ATT	2.03	1.86	0.17	-0.04	0.19
Probability of treatment seeking for an illness in the last one month	Model 1	Unmatched	74.3	67.0	7.3%		
		ATT	73.3	65.2	8.1	7.9	12.0
	Model 2	Unmatched	74.3	61.0	13.3		
		ATT	73.3	65.9	7.4	6.1	10.6
% of households with at least one IPD admission in the past 1 year	Model 1	Unmatched	0.061	0.08	-0.019		
		ATT	0.06	0.079	-0.019	-4.8	0.9
	Model 2	Unmatched	0.061	0.057	0.004		
		ATT	0.065	0.058	0.007	-2.1	4.1
OOP health payment (direct medical spending)	Model 1	Unmatched	1610.2	2178.3	-568.1		
		ATT	1567.8	2179.9	-612.2	-1141.5	-59.4
	Model 2	Unmatched	1610.2	2423.9	-813.7		
		ATT	1596.9	2834.7	-1237.8	-1675.0	-183.5
OOP non-medical spending (in birr)	Model 1	Unmatched	279.6	196.9	82.7		
		ATT	272.6	205	67.7	-27.0	173.1
	Model 2	Unmatched	279.6	140.6	139		
		ATT	261	142	119	45.9	230.0
Total OOP spending (in birr)	Model 1	Unmatched	1889.8	2375.2	-485.4		
		ATT	1840.4	2384.9	-544.5	-1111.5	56.8
	Model 2	Unmatched	1889.8	2564.5	-674.7		
		ATT	1857.9	2976.7	-1118.8	-1067.2	-340.2
% of households with catastrophic health expenditure^b^	Model 1	Unmatched	6.4	8.7	-2.3		
		ATT	6.4	9.5	-3.1	-5.98	-0.38
	Model 2	Unmatched	6.4	9.9	-3.5		
		ATT	6.3	11.8	-5.5	-7.48	-0.85
% of households falling below poverty line after OOP health payment	Model 1	Unmatched	1.39	1.02	0.37		
		ATT	1.4	1.2	0.2	-0.74	1.18
	Model 2	Unmatched	1.4	2.2	-0.8		
		ATT	1.3	3.1	-1.8	-3.42	0.14
Increase in poverty gap after OOP health payment	Model 1	Unmatched	0.51	0.48	0.03		
		ATT	0.51	0.62	-0.11	-0.45	0.27
	Model 2	Unmatched	0.51	0.80	-0.29		
		ATT	0.54	0.85	-0.31	-0.73	0.03

^a^Comparison groups for model 1 and model 2 were non-members in CBHI and non-CBHI woredas, respectively

^b^Catastrophic health expenditure: household’s annual direct medical cost > 10% of total expenditure

CBHI membership also significantly reduced annual OOP health expenditure. CBHI member households had ETB 545 (23%) and ETB 1119 (38%) lower total annual OOP spending compared to non-members from CBHI and non-CBHI woredas, respectively. Reduction in direct medical spending was the source of reduced OOP among members. On the other hand, member households had slightly higher non-medical spending compared to non-members. This may be a result of more frequent health facility visits among members leading to a higher non-medical cost of healthcare that is not covered by CBHI (Table 5).

The proportion of households with catastrophic health expenditure—defined as annual direct medical expenses totaling more than 10% of the household’s total expenditure, decreased by 3.1% and 5.5% compared to those among non-members from CBHI and non-CBHI woredas, respectively (Table 5).

## Discussion

Studies from Ethiopia [22–26], other African Countries [34–36], and Asia [37–40] have examined the impacts achieved through CBHI with respect to access to needed care and financial protection. Synthesis of these studies and systematic reviews focusing on low- and middle-income countries [41–51] showed moderate impact of CBHI on utilization of health care among beneficiaries and financial protection for its members. Moreover, effects were mostly marginal, context dependent, and not reproduced in some of the studies [40, 46].

Community-based health insurance is increasingly being adopted as an alternative healthcare financing strategy in low- and middle-income countries, including Ethiopia. In Ethiopia, it has been scaled-up since 2015 [18, 19]. Despite rapid expansion in its implementation, evidence of effectiveness have been largely limited to the pilot phase of the program and/or very small geographic coverage [22–24, 26]. The current study provided comprehensive evidence on the effect CBHI among its members in relation to health service utilization and financial risk protection by employing a more rigorous design and analysis involving PSM, which was instrumental in addressing the prevailing challenge of selection bias in previous studies. Matched comparison between CBHI members and non-members indicated that CBHI membership is positively and significantly associated with a number of health service utilization indicators while it is negatively and significantly associated with the incidence of catastrophic health expenditures.

Utilization of health services, measured in terms of both treatment seeking during illness and average annual per capita OPD visits, was in general higher in this study compared to reports from the routine health information system. The 2020 edition of Ethiopia’s Health and Health-Related Indicators reported annual outpatient attendance per capita of 1.02 [52], which was twice higher compared to the 2015 level of 0.48 visits per person per year [53] but half of the 2020 target set in the Health Sector Transformation Plan [54]. In our study, per capita annual OPD visit was 1.77 visits per person per year. Health service utilization among CBHI member households is even higher compared to that of non-members from CBHI and non-CBHI woredas. Annual number of OPD visits per capita among CBHI members was 2.09 OPD visits per capita per year. PSM estimates indicated that CBHI members had 22.6% and 9.1% higher OPD visits compared to their matched non-member households from CBHI implementing and non-CBHI implementing woredas, respectively. Similarly, healthcare seeking among sick household members was higher among CBHI members compared to non-members, even though the difference was smaller. The probability of treatment seeking among household members with an illness was 70% among CBHI members compared to 67% among non-members from CBHI woredas and 58% among households from non-CBHI woredas.

The impact of CBHI on per capita OPD visit as estimated in this study was lower compared to findings from the evaluation of the pilot study which reported 45–64% increase in frequency of visits [23]. The positive impact of CBHI on health service utilization in general corroborated sub-national studies in Ethiopia [25], and similar studies from elsewhere [44, 47, 55]. The relatively lower effect size we identified compared to findings from the evaluation of the pilot project in the early years [23] could be a result of differences between intensity of implementation of CBHI schemes between the pilot and scale-up phases. Moreover, the spillover effect of CBHI on the healthcare utilization patterns of non-members could also narrow the gap between members and non-members while health service utilization increases over time. Per capita OPD visit among individuals from CBHI member households was 0.15 in two months (equivalent to 0.9 visits per capita per year) in 2011 [23] compared to 2.09 visits per capita per year in the current study.

A possible mechanism for the observed positive impact of CBHI on health service utilization in Ethiopia was removal of financial barriers to health service use. Previous national studies reported financial barrier as one of the common causes of not using needed health services among Ethiopian households [6]. Similar findings were observed in the current study; however, the role of financial barriers was lower among CBHI member households. Financial barriers were a reason for not seeking health care among sick individuals in 35.4% of cases among CBHI members compared to 64.3% and 56.1% among non-members from CBHI and non-CBHI woredas, respectively. These findings are indicative of the positive role of CBHI in eliminating financial barrier to the use of health services.

Ensuring financial protection is one of the strategic areas of the health sector [18, 31]. CBHI is one of the major healthcare financing strategies introduced to ensure financial protection among households in the informal sector [18]. In this study, we found evidence supporting the claim that CBHI improves financial protection. CBHI has led to reduced OOP health expenditure and protected insured households from catastrophic health expenditure. From the descriptive results, we found that the average annual OOP health expenditure among CBHI member households was half that of households from non-CBHI woredas and two thirds that of non-CBHI members from CBHI woredas. Our findings show that, on average, a CBHI member household incurs an OOP payment of 612 birr and 1238 birr less in direct medical costs compared to non-members from CBHI and non-CBHI woredas, respectively. However, the reverse is true when direct non-medical costs are considered. The direct non-medical spending of CBHI members is higher compared to that of non-members. This is expected as CBHI membership increases utilization of health services, which incurs direct non-medical expenses which are not covered by the scheme. PSM estimates also confirmed that CBHI membership has resulted in a 28% to 43% reduction in annual OOP payments. A similar finding was also reported by the evaluation of the pilot phase even though the magnitude of effect was not comparable because of methodological differences [50].

As a result of reduced OOP health expenditure, the incidence of catastrophic health expenditure among CBHI members is much lower than that of non-members. This result is in line with the key objective of health insurance – to provide financial protection and minimize the extent to which households incur catastrophic health expenditures due to health care payments. CBHI member households were 32.6% to 46.6% less likely to incur catastrophic health expenditures compared to non-members. These results were consistent with findings from a sub-national study in Ethiopia [22, 23] and other studies examining the impacts of CBHI with comparable benefit packages [36, 37, 41] while it contradicts some studies that reported no or negative impacts of CBHI schemes [40].

Although the CBHI scheme plays a substantial role in reducing OOP payments and protecting households from catastrophic expenditures, the results reveal that the proportion of CBHI member households being pushed into poverty due to health care payments is significantly higher than that of non-members from CBHI woredas and lower than that of households from non-CBHI woredas. This pattern may be in part attributed to a higher incidence of CBHI uptake among the poorest households. The fact that the mean normalized positive net poverty gap is lower among members (1.5%) compared to non-members (2.1%), as well as the generally lower OOP health spending among CBHI members, also suggests that the pattern in the rate of impoverishment is driven by the lower socio-economic status of CBHI member households. Our finding showed that households in the lowest quintile of expenditure constitute 21% of CBHI members compared to 16–17% of non-member households. Administrative data also indicates that in 2020, about 21.2% of CBHI members were indigents [19].

This study examined several dimensions of the effect of CBHI. Potential bias in the estimation of the impact of CBHI is potentially a problem because of self-selection, instead of randomization, to CBHI membership. In order to overcome potential threats to validity because of self-selection, multiple non-exposed groups were used in the study design, and propensity score matching was applied for the analyses. PSM allowed us to balance most of the observed covariates in the matched sample of the insured and uninsured groups. This makes the impact estimates more robust and more precise than they would have been if the analysis did not account for self-selection.

Recall bias is another potential limitation. However, we have no reason to believe that recall bias affected the estimates differently between CBHI member and non-member respondents. As a result, we believe any possible underestimation of illness or health service use did not affect the effect sizes significantly. Most of the analysis in the estimation of the impact of CBHI relied on the one-month recall period to overcome issues with recall.

Consumption and expenditure data were collected during a fasting season for most of the study sites. This might have led us to underestimate consumption and households’ capacity to pay which in turn might have led us to overestimate the incidence of catastrophic and impoverishing health expenditures. However, we do not have a reason to think that these possible biases would vary between CBHI members and non-members.

## Conclusions

This study provides evidence of a positive effect of community-based health insurance in Ethiopia. CBHI membership increased the utilization of health services and reduced the incidence of catastrophic health expenditure. CBHI should be strengthened as an important strategy for promoting universal health coverage from the perspectives of increasing health service utilization, promoting equitable health services, and ensuring financial protection among households in the informal sector. CBHI should be scaled up to all woredas and membership increased among already implementing woredas to cover all households in the informal sector.

## Supplementary Information

**Additional file 1.**

## Data Availability

The datasets analyzed during the current study are available from the corresponding author on reasonable request.

## Abbreviations

ATT: Average treatment effect on the treated
CBHI: Community-based health insurance
CSPro: Census and Survey Processing System
EA: Enumeration area
OPE: Outpatient department
OOP: Out-of-pocket
PSM: Propensity score matching
SNNP: Southern Nations Nationalities and Peoples
THE: Total health expenditure
UHC: Universal Health Coverage
US$: United States Dollars
