# Supplementary material for: The impact of community-based health insurance on health service utilization and financial risk protection in Ethiopia
Source: BMC Health Serv Res. 2023 Jan 23;23:67. doi: 10.1186/s12913-022-09019-6 (PMC9869550; doi:10.1186/s12913-022-09019-6)
Supplement: Supplementary file 1 — Additional file 1. [file 12913_2022_9019_MOESM1_ESM.docx]

**Supplementary file**

**Ref**: Submission ID 49cbc014-2922-40e6-bdba-4779de40b294

**Title**: "The impact of community-based health insurance on health service utilization and financial risk protection in Ethiopia"

**Description**: This supplementary file presents indicators of the quality of matching achieved from propensity score matching method that was applied to account for the issue of self-selection to enroll in CBHI schemes. There were two models constructed under this study; quality of matching is reported separately for the two models as follows. In addition, we also presented the outputs of models that were used to calculate propensity scores.

1. **Model 1 (Insured vs. uninsured in CBHI districts)**
   1. **Quality of matching**

Table 1. Common support (Model 1)

|  | **Common support** | | |
| --- | --- | --- | --- |
| Treatment assignment | Off Support | On Support | Total |
| Untreated | 132 | 1,731 | 1,863 |
| Treated | 45 | 1,541 | 1,586 |
| **Total** | **177** | **3,272** | **3,449** |

Figure 1. propensity score histogram by treatment status (Model 1)

Table 2. Additional quality of matching tests (Model 1)

| **Variable** | **Unmatched** | **Mean** | |  | **%reduct** | **t-test** | |  |
| --- | --- | --- | --- | --- | --- | --- | --- | --- |
|  | **Matched** | **Treated** | **Control** | **%bias** | **\|bias\|** | **t** | **p>t** | **V(T)/V(C)** |
| Sex_Male | U | 0.78562 | 0.75953 | 6.2 |  | 1.82 | 0.069 | . |
|  | M | 0.78196 | 0.79299 | -2.6 | 57.7 | -0.75 | 0.454 | . |
| Marital status_Married | U | 0.78941 | 0.7402 | 11.6 |  | 3.39 | 0.001 | . |
|  | M | 0.78456 | 0.80013 | -3.7 | 68.3 | -1.07 | 0.287 | . |
| Literacy_Leterate | U | 0.52207 | 0.56575 | -8.8 |  | -2.57 | 0.01 | . |
|  | M | 0.51071 | 0.51006 | 0.1 | 98.5 | 0.04 | 0.971 | . |
| Education_Primary | U | 0.23897 | 0.21739 | 5.1 |  | 1.51 | 0.132 | . |
|  | M | 0.2401 | 0.25243 | -2.9 | 42.9 | -0.79 | 0.427 | . |
| Education_Secondary and above | U | 0.20996 | 0.31347 | -23.7 |  | -6.9 | 0 | . |
|  | M | 0.2122 | 0.20247 | 2.2 | 90.6 | 0.67 | 0.505 | . |
| Occupation_Self employed | U | 0.78562 | 0.70639 | 18.3 |  | 5.33 | 0 | . |
|  | M | 0.7852 | 0.78845 | -0.7 | 95.9 | -0.22 | 0.826 | . |
| Occupation_Employed (Gov and private) | U | 0.04414 | 0.14117 | -33.9 |  | -9.75 | 0 | . |
|  | M | 0.04283 | 0.03958 | 1.1 | 96.7 | 0.45 | 0.651 | . |
| Occupation_Others | U | 0.02837 | 0.03757 | -5.2 |  | -1.5 | 0.134 | . |
|  | M | 0.0292 | 0.0292 | 0 | 100 | 0 | 1 | . |
| Household size | U | 5.1003 | 4.2984 | 36 |  | 10.53 | 0 | 1.01 |
|  | M | 5.0149 | 5.0123 | 0.1 | 99.7 | 0.03 | 0.974 | 0.87* |
| Age group_36 to 45 | U | 0.27554 | 0.23027 | 10.4 |  | 3.06 | 0.002 | . |
|  | M | 0.28358 | 0.28812 | -1 | 90 | -0.28 | 0.78 | . |
| Age group_46 to 55 | U | 0.31967 | 0.20988 | 25.1 |  | 7.38 | 0 | . |
|  | M | 0.30759 | 0.30175 | 1.3 | 94.7 | 0.35 | 0.725 | . |
| Age group_56 and above | U | 0.1425 | 0.10467 | 11.5 |  | 3.39 | 0.001 | . |
|  | M | 0.14212 | 0.14731 | -1.6 | 86.3 | -0.41 | 0.682 | . |
| Self-reported health status_Good | U | 0.43064 | 0.40419 | 5.4 |  | 1.57 | 0.116 | . |
|  | M | 0.43024 | 0.44906 | -3.8 | 28.9 | -1.05 | 0.293 | . |
| Self-reported health status_Moderate | U | 0.19168 | 0.15298 | 10.3 |  | 3.01 | 0.003 | . |
|  | M | 0.18819 | 0.17975 | 2.2 | 78.2 | 0.6 | 0.546 | . |
| Self-reported health status_Bad | U | 0.03972 | 0.0424 | -1.4 |  | -0.4 | 0.693 | . |
|  | M | 0.04088 | 0.04283 | -1 | 27.4 | -0.27 | 0.787 | . |
| Self-reported health status_Very bad | U | 0.00631 | 0.00644 | -0.2 |  | -0.05 | 0.96 | . |
|  | M | 0.00649 | 0.00324 | 4.1 | -2285 | 1.29 | 0.196 | . |
| Per capita expenditure quintiles_2nd quintile | U | 0.2087 | 0.18089 | 7 |  | 2.06 | 0.039 | . |
|  | M | 0.2109 | 0.19663 | 3.6 | 48.7 | 0.98 | 0.325 | . |
| Per capita expenditure quintiles_3rd quintile | U | 0.23518 | 0.1986 | 8.9 |  | 2.61 | 0.009 | . |
|  | M | 0.23297 | 0.24335 | -2.5 | 71.6 | -0.68 | 0.499 | . |
| Per capita expenditure quintiles_4th quintile | U | 0.19357 | 0.21739 | -5.9 |  | -1.72 | 0.085 | . |
|  | M | 0.19598 | 0.21934 | -5.8 | 1.9 | -1.6 | 0.11 | . |
| Per capita expenditure quintiles_5th quintile | U | 0.14943 | 0.23994 | -23 |  | -6.68 | 0 | . |
|  | M | 0.1512 | 0.12395 | 6.9 | 69.9 | 2.2 | 0.028 | . |
| Chronic illness | U | 0.17844 | 0.13688 | 11.4 |  | 3.36 | 0.001 | . |
|  | M | 0.17262 | 0.14666 | 7.1 | 37.5 | 1.97 | 0.049 | . |
| Religion_Muslim | U | 0.36129 | 0.29844 | 13.4 |  | 3.93 | 0 | . |
|  | M | 0.35756 | 0.37508 | -3.7 | 72.1 | -1.01 | 0.313 | . |
| Religion_Others | U | 0.01009 | 0.0059 | 4.7 |  | 1.39 | 0.165 | . |
|  | M | 0.01038 | 0.00389 | 7.3 | -55.1 | 2.14 | 0.032 | . |

- 1. **Model output**

Table 3. Association of CBHI membership with socio-demographic characteristics of respondents: Probit regression output (Model 1)

| Association of CBHI membership with sociodemographic characteristics of study households | | | | | | |
| --- | --- | --- | --- | --- | --- | --- |
| Probit regression | Number of obs | = | 3,449 |  |  |  |
|  | LR chi2(23) | = | 294.26 |  |  |  |
|  | Prob > chi2 | = | 0 |  |  |  |
| Log likelihood = -2232.3971 | Pseudo R2 | = | 0.0618 |  |  |  |
|  |  |  |  |  |  |  |

| **Covariates** | **Coef.** | **Std. Err.** | **z** | **P>z** | **[95% CI]** | |
| --- | --- | --- | --- | --- | --- | --- |
| Sex_Male | -0.05153 | 0.076519 | -0.67 | 0.501 | -0.20151 | 0.098443 |
| Marital status_Married | 0.069598 | 0.07746 | 0.9 | 0.369 | -0.08222 | 0.221416 |
| Literacy_Leterate | 0.347051 | 0.1042 | 3.33 | **0.001** | 0.14282 | 0.551279 |
| Educational status |  |  |  |  |  |  |
| Primary education | -0.27154 | 0.109494 | -2.48 | **0.013** | -0.48614 | -0.05694 |
| Secondary and above | -0.27733 | 0.112967 | -2.45 | **0.014** | -0.49875 | -0.05592 |
| Occupation (Ref: Unemployed) |  |  |  |  |  |  |
| Self-employed (mostly Farmers) | -0.0908 | 0.075893 | -1.2 | 0.232 | -0.23954 | 0.05795 |
| Employed (private and public) | -0.64343 | 0.111293 | -5.78 | **0** | -0.86156 | -0.4253 |
| Other Occupation | -0.29617 | 0.140522 | -2.11 | **0.035** | -0.57158 | -0.02075 |
| Household size | 0.063909 | 0.012935 | 4.94 | **0** | 0.03856 | 0.089262 |
| Age group of head (Ref: <=35) |  |  |  |  |  |  |
| 36-45 years | 0.286868 | 0.06202 | 4.63 | **0** | 0.16531 | 0.408424 |
| 46-64 years | 0.437932 | 0.063614 | 6.88 | **0** | 0.31325 | 0.562613 |
| 65 years or older | 0.427612 | 0.084548 | 5.06 | **0** | 0.2619 | 0.593323 |
| Self-reported health status (Ref: Very good) |  |  |  |  |  |  |
| Good | 0.056401 | 0.050983 | 1.11 | 0.269 | -0.04352 | 0.156326 |
| moderate | 0.047268 | 0.070906 | 0.67 | 0.505 | -0.09171 | 0.186241 |
| bad | -0.1514 | 0.125038 | -1.21 | 0.226 | -0.39647 | 0.093667 |
| very bad | -0.11167 | 0.279312 | -0.4 | 0.689 | -0.65911 | 0.435773 |
| Expenditure quintile (Ref: Poorest) |  |  |  |  |  |  |
| Poorer | -0.00882 | 0.07147 | -0.12 | 0.902 | -0.1489 | 0.131257 |
| Middle | 0.053453 | 0.071426 | 0.75 | 0.454 | -0.08654 | 0.193446 |
| Richer | -0.01116 | 0.075466 | -0.15 | 0.882 | -0.15907 | 0.136754 |
| Richest | -0.02756 | 0.084847 | -0.32 | 0.745 | -0.19386 | 0.138735 |
| Chronic illness (% of household members) | 0.149956 | 0.068944 | 2.18 | **0.03** | 0.01483 | 0.285083 |
| Religion (Ref: Christian) |  |  |  |  |  |  |
| Musilim | 0.149437 | 0.047513 | 3.15 | **0.002** | 0.05631 | 0.242561 |
| Others | 0.444516 | 0.246425 | 1.8 | **0.071** | -0.03847 | 0.9275 |
| _cons | -0.68081 | 0.117892 | -5.77 | **0** | -0.91187 | -0.44974 |

1. **Model 2 (Insured vs. uninsured in non-CBHI districts)**
   1. **Quality of matching**

Table 4. Common support (Model 2)

|  | **Common support** | | |
| --- | --- | --- | --- |
| Treatment assignment | Off Support | On Support | Total |
| Untreated | 141 | 648 | 789 |
| Treated | 306 | 1,280 | 1,586 |
| Total | 447 | 1,928 | 2,375 |

Figure 2. propensity score histogram by treatment status (Model 2)

Table 5. Additional quality of matching tests (Model 2)

| **Variable** | **Unmatched** | **Mean** | |  | **%reduct** | **t-test** | |  |
| --- | --- | --- | --- | --- | --- | --- | --- | --- |
|  | **Matched** | **Treated** | **Control** | **%bias** | **\|bias\|** | **t** | **p>t** | **V(T)/V(C)** |
| Sex_Male | U | 0.78562 | 0.70976 | 17.5 |  | 4.09 | 0 | . |
|  | M | 0.77422 | 0.76172 | 2.9 | 83.5 | 0.75 | 0.454 | . |
| Marital status_Married | U | 0.78941 | 0.69075 | 22.6 |  | 5.31 | 0 | . |
|  | M | 0.77734 | 0.77109 | 1.4 | 93.7 | 0.38 | 0.705 | . |
| Literacy_Leterate | U | 0.52207 | 0.61343 | -18.5 |  | -4.23 | 0 | . |
|  | M | 0.52031 | 0.54297 | -4.6 | 75.2 | -1.15 | 0.251 | . |
| Education_Primary | U | 0.23897 | 0.17744 | 15.2 |  | 3.42 | 0.001 | . |
|  | M | 0.24219 | 0.24922 | -1.7 | 88.6 | -0.41 | 0.68 | . |
| Education_Secondary and above | U | 0.20996 | 0.40558 | -43.3 |  | -10.27 | 0 | . |
|  | M | 0.22187 | 0.21406 | 1.7 | 96 | 0.48 | 0.632 | . |
| Occupation_Self employed | U | 0.78562 | 0.59949 | 41.2 |  | 9.74 | 0 | . |
|  | M | 0.77656 | 0.78438 | -1.7 | 95.8 | -0.48 | 0.633 | . |
| Occupation_Employed (Gov and private) | U | 0.04414 | 0.18885 | -46.3 |  | -11.81 | 0 | . |
|  | M | 0.05156 | 0.04531 | 2 | 95.7 | 0.74 | 0.462 | . |
| Occupation_Others | U | 0.02837 | 0.04943 | -10.9 |  | -2.62 | 0.009 | . |
|  | M | 0.02969 | 0.01797 | 6.1 | 44.3 | 1.94 | 0.052 | . |
| Household size | U | 5.1003 | 4.2357 | 38.5 |  | 8.85 | 0 | 0.98 |
|  | M | 5.0031 | 5.2195 | -9.6 | 75 | -2.33 | 0.02 | 0.78* |
| Age group_36 to 45 | U | 0.27554 | 0.23447 | 9.4 |  | 2.14 | 0.032 | . |
|  | M | 0.27813 | 0.27969 | -0.4 | 96.2 | -0.09 | 0.93 | . |
| Age group_46 to 55 | U | 0.31967 | 0.23574 | 18.8 |  | 4.25 | 0 | . |
|  | M | 0.30625 | 0.31875 | -2.8 | 85.1 | -0.68 | 0.495 | . |
| Age group_56 and above | U | 0.1425 | 0.109 | 10.1 |  | 2.28 | 0.023 | . |
|  | M | 0.13984 | 0.11953 | 6.1 | 39.4 | 1.53 | 0.126 | . |
| Self-reported health status_Good | U | 0.43064 | 0.46008 | -5.9 |  | -1.36 | 0.174 | . |
|  | M | 0.42734 | 0.45391 | -5.3 | 9.8 | -1.35 | 0.176 | . |
| Self-reported health status_Moderate | U | 0.19168 | 0.15589 | 9.4 |  | 2.14 | 0.032 | . |
|  | M | 0.19141 | 0.19687 | -1.4 | 84.7 | -0.35 | 0.727 | . |
| Self-reported health status_Bad | U | 0.03972 | 0.04309 | -1.7 |  | -0.39 | 0.696 | . |
|  | M | 0.04219 | 0.05703 | -7.4 | -340.5 | -1.73 | 0.084 | . |
| Self-reported health status_Very bad | U | 0.00631 | 0.00253 | 5.7 |  | 1.22 | 0.222 | . |
|  | M | 0.00625 | 0.00547 | 1.2 | 79.3 | 0.26 | 0.796 | . |
| Per capita expenditure quintiles_2nd quintile | U | 0.2087 | 0.1673 | 10.6 |  | 2.4 | 0.016 | . |
|  | M | 0.19687 | 0.21563 | -4.8 | 54.7 | -1.17 | 0.241 | . |
| Per capita expenditure quintiles_3rd quintile | U | 0.23518 | 0.14322 | 23.6 |  | 5.26 | 0 | . |
|  | M | 0.22734 | 0.25469 | -7 | 70.3 | -1.62 | 0.106 | . |
| Per capita expenditure quintiles_4th quintile | U | 0.19357 | 0.20786 | -3.6 |  | -0.82 | 0.411 | . |
|  | M | 0.19531 | 0.20313 | -1.9 | 45.3 | -0.49 | 0.621 | . |
| Per capita expenditure quintiles_5th quintile | U | 0.14943 | 0.30925 | -38.7 |  | -9.29 | 0 | . |
|  | M | 0.16094 | 0.12969 | 7.6 | 80.4 | 2.24 | 0.025 | . |
| Chronic illness | U | 0.17844 | 0.15082 | 7.4 |  | 1.69 | 0.091 | . |
|  | M | 0.18359 | 0.17656 | 1.9 | 74.5 | 0.46 | 0.644 | . |
| Religion_Muslim | U | 0.36129 | 0.13815 | 53.3 |  | 11.63 | 0 | . |
|  | M | 0.31016 | 0.28906 | 5 | 90.5 | 1.16 | 0.244 | . |
| Religion_Others | U | 0.01009 | 0.06717 | -29.9 |  | -7.9 | 0 | . |
|  | M | 0.00391 | 0.01094 | -3.7 | 87.7 | -2.07 | 0.038 | . |

- 1. **Model output**

Table 6. Association of CBHI membership with socio-demographic characteristics of respondents: Probit regression output (Model 2)

| Probit regression | Number of obs | = | 2,375 |
| --- | --- | --- | --- |
|  | LR chi2(23) | = | 436.48 |
|  | Prob > chi2 | = | 0 |
| Log likelihood = -1291.6246 | Pseudo R2 | = | 0.1445 |

| **Covariates** | **Coef.** | **Std. Err.** | **z** | **P>z** | **[95% CI]** | |
| --- | --- | --- | --- | --- | --- | --- |
| Sex_Male | 0.015814 | 0.094732 | 0.17 | 0.867 | -0.16986 | 0.201 |
| Marital status_Married | 0.162966 | 0.095267 | 1.71 | 0.087 | -0.02375 | 0.35 |
| Literacy_Leterate | 0.241489 | 0.139193 | 1.73 | 0.083 | -0.03132 | 0.514 |
| Educational status |  |  |  |  |  |  |
| Primary education | -0.30182 | 0.145747 | -2.07 | 0.038 | -0.58748 | -0.016 |
| Secondary and above | -0.51392 | 0.146378 | -3.51 | 0 | -0.80082 | -0.227 |
| Occupation (Ref: Unemployed) |  |  |  |  |  |  |
| Self-employed (mostly Farmers) | 0.20252 | 0.090259 | 2.24 | 0.025 | 0.025616 | 0.379 |
| Employed (private and public) | -0.51219 | 0.128621 | -3.98 | 0 | -0.76429 | -0.26 |
| Other Occupation | -0.24224 | 0.16626 | -1.46 | 0.145 | -0.5681 | 0.084 |
| Household size | 0.036663 | 0.016755 | 2.19 | 0.029 | 0.003823 | 0.07 |
| Age group of head (Ref: <=35) |  |  |  |  |  |  |
| 36-45 years | 0.205978 | 0.080093 | 2.57 | 0.01 | 0.048999 | 0.363 |
| 46-64 years | 0.266826 | 0.081221 | 3.29 | 0.001 | 0.107636 | 0.426 |
| 65 years or older | 0.281232 | 0.108908 | 2.58 | 0.01 | 0.067775 | 0.495 |
| Self-reported health status (Ref: Very good) |  |  |  |  |  |  |
| Good | -0.18191 | 0.067456 | -2.7 | 0.007 | -0.31412 | -0.05 |
| moderate | -0.09572 | 0.093112 | -1.03 | 0.304 | -0.27822 | 0.087 |
| bad | -0.16617 | 0.159793 | -1.04 | 0.298 | -0.47936 | 0.147 |
| very bad | 0.440846 | 0.441376 | 1 | 0.318 | -0.42424 | 1.306 |
| Expenditure quintile (Ref: Poorest) |  |  |  |  |  |  |
| Poorer | 0.026695 | 0.094414 | 0.28 | 0.777 | -0.15835 | 0.212 |
| Middle | 0.169106 | 0.096852 | 1.75 | 0.081 | -0.02072 | 0.359 |
| Richer | -0.04166 | 0.099606 | -0.42 | 0.676 | -0.23689 | 0.154 |
| Richest | -0.19531 | 0.108398 | -1.8 | 0.072 | -0.40777 | 0.017 |
| Chronic illness (% of household members) | 0.043732 | 0.084679 | 0.52 | 0.606 | -0.12224 | 0.21 |
| Religion (Ref: Christian) |  |  |  |  |  |  |
| Muslim | 0.627764 | 0.069653 | 9.01 | 0 | 0.491247 | 0.764 |
| Others | -1.26518 | 0.172999 | -7.31 | 0 | -1.60425 | -0.926 |
| _cons | -0.04278 | 0.150742 | -0.28 | 0.777 | -0.33823 | 0.253 |
